# Editorial: Unravelling Aβ toxicity: implications for Alzheimer's cognitive and behavioral deficits

**DOI:** 10.3389/fnagi.2026.1848776

**Published:** 2026-04-23

**Authors:** Md. Golam Sharoar, Neeraj Singh, Xiang-you Hu

**Affiliations:** 1 Alzheimer's Disease Research Program, Corewell Health Research Institute, Royal Oak, MI, United States; 2 Department of Internal Medicine, Oakland University William Beaumont School of Medicine, Rochester, MI, United States; 3 Department of Physiological Sciences, Oklahoma State University, Stillwater, OK, United States; 4 Department of Neuroscience, University of Connecticut Health, Farmington, CT, United States

**Keywords:** aging, Alzheimer's disease (AD), amyloid-β (Aβ) oligomers, cognitive impairment, GABAergic interneurons, neural circuit dysfunction, neuroinflammation, plaque burden

## 1 Introduction

Alzheimer's disease (AD) has long been associated with the accumulation of amyloid-β (Aβ) plaques and tau pathology; however, increasing evidence indicates that soluble Aβ oligomers, rather than insoluble plaques, are the primary drivers of neurotoxicity and early cognitive decline (Walsh and Selkoe, 2007; Selkoe and Hardy, 2016; Benilova et al., 2012). These oligomeric assemblies are highly dynamic, diffusible, and biologically active, capable of disrupting synaptic function, impairing long-term potentiation, and altering neuronal signaling well before overt neurodegeneration becomes apparent.

This shift from plaque-centric to oligomer-centric perspectives has profound implications for understanding AD. Aβ oligomers act as potent synaptotoxins, targeting neuronal receptors, perturbing calcium homeostasis, and inducing oxidative stress and inflammatory responses (Haass and Selkoe, 2007; De Strooper and Karran, 2016). Importantly, their levels correlate more closely with disease severity and cognitive impairment than plaque burden, reinforcing their central role in pathogenesis.

## 2 Research Topic content

The Frontiers Research Topic “*Unravelling A*β *toxicity: implications for Alzheimer's cognitive and behavioral deficits”* builds upon this evolving framework by examining how oligomeric Aβ interacts with aging, neuroinflammation, and neural circuit dynamics to drive disease progression. The contributions within this Research Topic emphasize that Aβ oligomer toxicity is not an isolated event but a context-dependent process shaped by cellular vulnerability, molecular diversity, and system-level responses. In this editorial, we synthesize these findings to highlight a shift toward a systems-level understanding of oligomer-driven toxicity, linking molecular mechanisms to cognitive and behavioral manifestations in AD.

### 2.1 Aging as a critical determinant of Aβ toxicity

A central theme emerging from this Research Topic is the role of aging as a permissive factor for Aβ-induced neuroinflammation and neuronal dysfunctions. Culley et al. demonstrate that acute exposure to Aβ oligomers produces markedly different outcomes depending on age. In aged animals, Aβ insult results in persistent cognitive deficits, synaptic loss, increased microglial activation, mitochondrial dysfunction, and propagation of tau pathology. In contrast, young brains exhibit resilience and recovery.

These findings suggest that aging transforms Aβ exposure from a transient insult into a self-sustaining pathological cascade. Mechanistically, age-related impairments in proteostasis, mitochondrial function, and immune regulation likely create a vulnerable environment that amplifies Aβ toxicity (Hou et al., 2019; Mattson and Arumugam, 2018). Thus, aging should be considered not merely a risk factor but a biological amplifier of disease progression.

### 2.2 From amyloid burden to neural circuit dysfunction and cognitive deficit

A critical insight in Alzheimer's disease (AD) is the weak relationship between amyloid plaque burden and cognitive impairment. Using the TgF344-AD rat model, Futácsi et al. show that cognitive decline correlates more strongly with glial activation and selective loss of GABAergic interneurons than with plaque load. Despite substantial amyloid deposition in both the hippocampus and medial prefrontal cortex, cognitive deficits were not directly linked to plaque burden, underscoring the role of inhibitory circuit dysfunction and network imbalance in AD pathology.

These findings align with the view that AD is primarily a disorder of synaptic and circuit-level dysfunction (Busche and Konnerth, 2016; Palop and Mucke, 2010), in which Aβ disrupts excitatory–inhibitory balance and neural communication. Complementary human data Pyun et al. in this Research Topic further indicate that reduced cerebrospinal fluid Aβ_1−−42_ mediates the relationship between cognitive impairment and psychosis, linking amyloid pathology to neuropsychiatric symptoms.

Notably, emerging therapeutic approaches targeting Aβ dynamics remain promising. Park et al. showed that delivery of a secreted Aβ-cleaving protease (SecNIa) via AAV reduced soluble and insoluble Aβ, decreased plaque burden, and improved cognition in 5xFAD mice. Together, these findings suggest that although amyloid accumulation alone does not fully account for cognitive decline, targeted modulation of Aβ can confer functional benefits, reinforcing a systems-level link between molecular pathology, circuit dysfunction, and clinical outcomes in AD.

### 2.3 Molecular diversity of Aβ and its functional implications

The Research Topic also underscores the heterogeneity of Aβ species and their distinct biological effects. Oligomeric Aβ is widely recognized as the most synaptotoxic form, capable of impairing synaptic plasticity, inducing oxidative stress, and disrupting neuronal signaling (Walsh and Selkoe, 2007; Benilova et al., 2012). However, Cavaleri challenges the exclusively pathogenic view of Aβ, proposing that certain forms may have physiological or protective roles, such as metal ion chelation. This duality suggests that Aβ toxicity arises not simply from its presence but from imbalances in its production, aggregation, and clearance, as well as from shifts in its molecular composition (Haass and Selkoe, 2007). Consequently, therapeutic strategies that indiscriminately remove Aβ may overlook important functional aspects and fail to target the most toxic species.

### 2.4 Emerging regulatory mechanisms: RNA and beyond

Beyond classical pathways, this Research Topic introduces novel regulatory mechanisms contributing to Aβ toxicity. A study in *Caenorhabditis elegans* (Alshareef et al.) reveal that age-associated circular RNAs (circRNAs) modulate Aβ-induced neurotoxicity, with loss of specific circRNAs mitigating pathological effects. These findings expand the landscape of AD research to include post-transcriptional regulation and non-coding RNA networks (Salta and De Strooper, 2017).

Such mechanisms highlight the complexity of Aβ toxicity, which is embedded within broader gene regulatory systems. Understanding these interactions may uncover new therapeutic targets that go beyond protein-centric approaches.

### 2.5 Therapeutic implications and future directions

The collective findings from this Research Topic provide important insights into why many anti-amyloid therapies have failed to achieve meaningful clinical outcomes. First, interventions are often initiated too late, after irreversible synaptic and circuit damage has occurred (Sperling et al., 2011). Second, many approaches do not distinguish between toxic and non-toxic forms of Aβ. Third, Aβ operates within a complex network of interacting processes, including aging, inflammation, and neuronal signaling (Benilova et al., 2012; Hou et al., 2019; Busche and Konnerth, 2016; Palop and Mucke, 2010; Leng and Edison, 2021).

Future therapeutic strategies should therefore adopt a multidimensional approach, targeting not only Aβ but also the downstream mechanisms that mediate its toxicity. Early intervention, modulation of neuroinflammation, protection of synaptic integrity, and enhancement of cellular resilience are likely to be critical components of effective treatment.

## 3 Conclusion

The studies presented in this Research Topic collectively advance a more nuanced understanding of AD. Aβ toxicity emerges as a dynamic and context-dependent process that disrupts neuronal function at multiple levels, from molecular interactions to neural circuits and behavior. Aging, neuroinflammation, and regulatory networks play essential roles in shaping this toxicity.

Moving forward, a shift from reductionist models toward integrative, systems-level frameworks will be essential for developing effective interventions. By linking molecular mechanisms to cognitive and behavioral outcomes, this body of work provides a foundation for rethinking AD and guiding future research and therapeutic innovation.
